# Exploring the Influence of Online Price Anchoring and Attribute Framing on the Likelihood of Hearing Aid Purchases

**DOI:** 10.3390/audiolres15020040

**Published:** 2025-04-12

**Authors:** Craig Richard St. Jean, Jacqueline Cummine, Gurjit Singh, William (Bill) Hodgetts

**Affiliations:** 1 Department of Communication Sciences and Disorders, University of Alberta, Edmonton, AB T6G 2G4, Canada; crstjean@ualberta.ca (C.R.S.J.); jcummine@ualberta.ca (J.C.); 2 Sonova Canada, Kitchener, ON N2E 1Y6, Canada; gurjit.singh@sonova.com; 3 Department of Psychology, Toronto Metropolitan University, Toronto, ON M5B 2K3, Canada; 4 Department of Speech-Language Pathology, University of Toronto, Toronto, ON M5G 1V7, Canada

**Keywords:** message framing, purchase intention, anchoring effect, teleaudiology, health psychology, hearing aids

## Abstract

**Background/Objectives**: This study investigated whether exposure to various types of information online can influence adults aged 40 and above in their likelihood to purchase hearing aids (HAs). Specifically, it examined the effects of price anchoring, using high or low HA prices in advertisements, and attribute framing, using product descriptions that highlighted lifestyle appeal or technological capabilities. **Methods**: In a 2 × 2 experimental design, 271 participants browsed a website simulating an online search for hearing health information. Participants then rated their likelihood of purchasing three fictitious HAs as well as their likelihood of not purchasing any device. **Results**: Two-way ANCOVAs indicated no significant main effects of anchoring or framing on purchase likelihood for the fictitious devices (covariates included self-rated hearing ability, trust in online health information, and HA knowledge and importance). No significant interaction effects emerged. However, exploratory analyses revealed significant anchoring effects for two of the three devices among participants with below-median self-rated hearing. Additionally, self-rated knowledge was a significant covariate in the model for all devices (*p* < 0.001), positively correlating with purchase likelihood. Participants with above-median self-rated knowledge showed significantly higher purchase likelihood for all devices (*p* < 0.001, *d* ≥ 0.572 for all comparisons), while those with below-median knowledge displayed a significant anchoring effect for two outcomes. **Conclusions**: Enhanced HA knowledge may increase HA purchase likelihood and reduce the potential anchoring effects of online advertising. Further research is needed to determine the impact of exposure to high or low prices on HA purchase decisions, especially among those with poorer hearing.

## 1. Introduction

Cost is one among a wide range of non-audiological factors that have been shown to play a role in whether individuals adopt hearing aids (HAs) [1]. A number of studies have reported that the high cost of the devices may be a barrier for many [2,3,4]. It has been estimated that just a third of the cost of HAs purchased through a clinic is for the devices themselves, with the remainder covering audiological services and clinic overheads [5]. Consequently, recent legislation allowing for the sale of over the counter (OTC) HAs in the United States has been heralded as a democratizing shift in the accessibility of personal amplification [6]. However, for individuals with more complex symptoms or more severe hearing loss, the services of an audiology clinic, and thus the bundled prices of their HAs, may remain necessary [7].

Many people experiencing hearing loss turn to the internet as the first response to their symptoms [8]. Conducting a Google search for ‘hearing aids’ will largely result in sponsored advertisements for OTC HAs [9]. These ads quote prices in the low hundreds of Canadian and US dollars, which are substantially lower than the prices a potential buyer would encounter at a licensed audiology clinic. A set of prescribed HAs may range in price between USD 2000 and USD 7000, depending on technology level and accompanying device and patient services, with an average cost estimated to be USD 4700 [10]. Google search trend data for the United States indicates that searches for ‘hearing aids’ vastly outnumber searches for ‘over the counter hearing aids’ in frequency [11]. This may lead to scenarios in which the low advertised prices might give internet users, unfamiliar with the distinction between OTC HAs and those dispensed by licensed clinics, an inaccurate idea of the typical cost of HAs. As the effect of exposure to high or low figures has been shown to influence judgements made up to a week later without the magnitude of these effects diminishing [12], the impact of exposure to OTC HA prices online may be of interest to healthcare providers as well as HA vendors and manufacturers.

### 1.1. Anchoring Effects

Encountering OTC HA prices at the beginning of one’s journey to hearing rehabilitation may lead to an anchoring effect: a situation in which a future prediction or decision is influenced by an initially available piece of information, or ‘anchor’, particularly in the absence of further information about the topic [13]. The anchor in this case is the initial piece of specific information provided before a judgment is made [14]. Initially proposed by Tversky and Kahneman [15], the anchoring effect is a widely acknowledged bias in the decision-making process and is one of the most frequently studied biases in controlled laboratory settings [16,17]. This phenomenon stems from the tendency of individuals to place undue emphasis on specific information when estimating a value as part of the decision-making process [18]. Essentially, when evaluating an ambiguous situation or object, most individuals begin their estimation with an available reference point and adjust their final judgment in relation to that point, even if the original value was entirely arbitrary [19]. In situations of uncertainty regarding product attributes or quality, consumers are inclined to base their attitudes and buying decisions on readily available information, such as advertised prices [20]. Accordingly, researchers have found stronger anchoring effects when the item being rated is less familiar or relevant to participants, alongside inverse relationships between participants’ confidence in their ratings and the extent to which ratings are swayed by an anchor [18,21]. To date, we are unaware of any studies that have investigated potential anchoring effects as part of the decision-making process when purchasing HAs.

### 1.2. Attribute Framing

In addition to sponsored advertisements touting low prices, prospective HA users looking for information online are also likely to encounter a variety of messaging strategies intended to make the available devices seem more attractive. The effects of message framing on attitudes and intentions toward hearing loss have been explored in a handful of studies; however, these studies have focused on comparing gain- or loss-framed messaging [22,23,24]. In advertising, consumers are more likely to encounter ‘attribute framing’ (sometimes called emphasis framing), which refers to choosing to place focus on one aspect of a product or issue versus another [25]. For consumer products, attribute framing may involve highlighting aspects of the devices’ technological capability and performance, or the lifestyle benefits that users will incur when using the product, or some combination of the two. To date, little research exists exploring the impact of attribute framing in relation to hearing health decision making. However, a recent study that investigated the use of medical language (vs. plainer language) about hearing loss and amplification in different contexts found that the use of medical language by family physicians may be predictive of more negative attitudes toward hearing loss but more positive attitudes toward HAs [26]. In contrast, another recent study found that the use of medical language in newspaper articles about hearing loss may be predictive of adverse attitudes toward the use of HAs [27].

A number of recent studies have explored the potential joint effects of attribute framing and anchoring. Wu and Cheng [28] contend that prices and attribute descriptions are the predominant forms of information prospective consumers will encounter when shopping online. The potential interaction between anchoring and message framing on consumers’ purchase intention and willingness to pay has been explored in several studies, which have yielded divergent results. Wu and Cheng [28] manipulated both factors in an experiment measuring willingness to pay and purchase intention for a fictitious language translation device. They reported a main effect for message framing on purchase intention and a main effect for anchoring on willingness to pay, as well as a significant interaction in which both purchase intention and willingness to pay were highest among participants who were exposed to a high anchor price coupled with a positively framed product description (“80% translation accuracy” compared with the negatively framed “20% error rate”). However, when the same analysis was applied after dividing the sample into groups with high and low subjective product knowledge, the interaction was only significant among the low-knowledge group [28]. Similarly, in an investigation of organic food purchase intention, Shan et al. [20] observed that participants with less knowledge of organic food were more susceptible to both anchoring and framing effects. They reported overall greater intentions toward purchasing organic food following exposure to a low price anchor and negatively framed messaging (highlighting loss incurred by not purchasing organic food), but without a significant interaction effect.

Andersson et al. [29] investigated the trade-offs between travel time and greenhouse gas emissions. They observed that individuals with higher self-rated levels of environmental concern were more strongly swayed by a high time anchor when asked how long they would be willing to extend a car trip to reduce CO_2_ emissions. Overall, they reported main effects for both anchoring and message framing, whereby participants were willing to extend their trips for longer following exposure to a high time anchor and a message about desired levels of personal CO_2_ emissions (versus an unrelated message about desired salt consumption), respectively, along with a significant interaction between these two conditions. Chen and Wang [30] investigated the effect of message framing (positively vs. negatively framed statements about the likelihood that housing prices would change) and price anchoring on real estate purchase intention and willingness to pay. They found no effect for framing but found significant effects for anchoring on purchase intention and willingness to pay, but in opposite directions: exposure to a high anchor decreased purchase intention but increased willingness to pay. While they observed that anchoring’s impact on purchase intention was moderated by the respondents’ level of optimism about the real estate market, they did not include measures of their knowledge of issue involvement in their analysis. In summary, the extant literature would suggest that attitudes towards products are influenced by framing effects, price anchors, and one’s familiarity with those products.

### 1.3. The Present Study

The combination of anchoring and framing effects has yet to be explored regarding the likelihood of HA purchase. The present study may contribute to a better understanding of online information presentation strategies which can increase or decrease purchase likelihood for HAs of varying levels of technology. Our findings may be of interest to parties who manufacture and/or market HAs or to consumers who are trying to navigate the complex amount of online information and targeted advertisements that permeate their online lives. Specifically, in the present study we asked: among individuals who read a webpage with information on HAs, will purchase likelihood for a selection of HA options differ as a function of exposure to the prices featured in a banner advertisement for HAs (low anchor—pricing suggestive of entry-level devices vs. high anchor—pricing suggestive of premium devices) and/or as a function of how the information about HAs on the webpage is framed (lifestyle appeal vs. technological capability)?

## 2. Materials and Methods

### 2.1. Design and Participants

We recruited 271 adults aged 40 or older on Amazon’s Mechanical Turk (MTurk) data collection platform. Participation was limited to MTurk users in the United States and Canada. Participants were randomly assigned to one of the four conditions in a 2 (price anchoring: low vs. high) by 2 (attribute framing: lifestyle benefit vs. technological capability) between-subjects design. Each participant received USD 1.00 as payment for completing the study, disbursed via their MTurk account. There were 69, 74, 64, and 64 participants, respectively, in the following groups: Group 1 (low anchor, lifestyle framing), Group 2 (low anchor, tech framing), Group 3 (high anchor, lifestyle framing), and Group 4 (high anchor, tech framing). The study received ethics approval from the University of Alberta’s Research Ethics Board.

### 2.2. Procedure and Experimental Manipulation

MTurk users who met the study’s inclusion criteria (resident of the United States or Canada; 40 years of age or older) saw our study advertised as ‘Online Hearing Health Information Study’, alongside the following description: “More and more people access health care information online, including information about hearing health and services for treating hearing loss. This study asks your impressions of information about hearing loss and types of hearing aids that are currently available”. Clicking the link to participate opened a new window in which users were taken to an experimental website developed by the research team.

**Figure 1:**
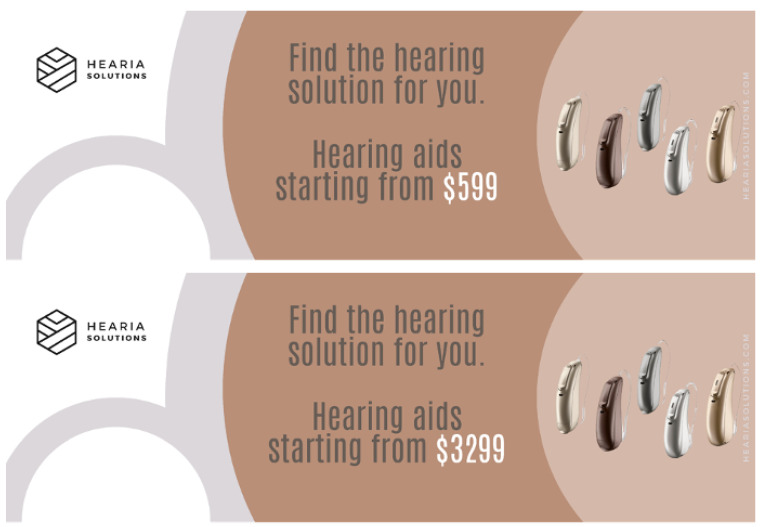
Banner advertisements seen in low (**top**) and high (**bottom**) anchor conditions.

Users who accessed the study first read an information letter about the tasks involved in the study and then completed an informed consent questionnaire, followed by a demographic information questionnaire. To simulate the experience of looking up information about hearing loss online, participants were instructed to read a page of text about hearing loss and its health impacts, after which they were asked to answer questions about the text at the bottom of the page. While reading, participants were exposed to our price anchoring manipulation, in which they were randomized to see banner advertisements for HAs that displayed either a high or low price point. The banners for the conditions were identical except for the price they quoted: they featured a logo for a fictitious hearing aid company named Hearia Solutions on the left and a collage of behind-the-ear HAs on the right. In the center, the banners read “Find the hearing solution for you”. In the low anchor condition, the banner additionally read “Hearing aids starting from USD 599”, while in the high anchor condition it read “Hearing aids starting from USD 3299”. The price’s text color was white to create contrast between it and the surrounding text. In each condition, the same banner was placed both at the top and bottom of the hearing loss information page to increase the likelihood that participants would notice it. The size and placement of the banner were designed to mimic common ways in which sponsored content banner advertisements are positioned on popular websites. See Figure 1 below for the banner advertisements used in the study.

The text among which the banners were embedded was four paragraphs in length, totaling 438 words, at a 16.6 Flesch–Kincaid reading level. It was written by the researchers and included a reference list. At the bottom of the page, participants used a visual analog scale to respond to the prompt “Using the slider below, tell us: How difficult was it to understand the information above?”. The left/right anchor points on the slider were “Very easy to understand (0)” and “Very difficult to understand (100)” and a number was displayed indicating where participants positioned the slider arrow along the line. Participants were then asked, “Approximately how many minutes did it take you to read the above information?” and entered their response in an open text field.

On the next page of the study, participants were asked to “Imagine that you needed a hearing aid. You look online to see what kinds of options are available and to learn about the range of prices. You visit a website for a leading hearing manufacturer. You read the following descriptions of their core line-up of hearing aids:” Below this text, participants were shown a table comparing technical specifications and prices of three fictitious hearing aid models, the QX50 (USD 1900), QX70 (USD 2600), and QX90 (USD 3300). The prices were chosen to represent approximate price ranges for entry-level, mid-range, and premium devices, respectively. In the ‘specifications’ column of the table, the technical features of each device were presented in a list format, with two features listed for the QX50, four listed for the QX70, and six listed for the QX90. In the ‘description’ column, participants were exposed to our message framing manipulation, in which they were randomized to see short descriptions of each model commenting on the same features, but with an emphasis either on benefits to the wearer’s daily activities (the ‘Lifestyle’ condition), or an emphasis on the device’s technological capabilities (the ‘Tech’ condition). The descriptions ranged between 46 words at the shortest (QX70 tech frame) and 65 words at the longest (QX90 lifestyle frame). The lifestyle-focused descriptions frequently used the words ‘you’ and ‘your’ to center the wearer in the description (e.g., “The QX70 is designed with your connected life in mind”.) while the tech-focused descriptions used impersonal, device-centered language (e.g., “The QX50 will help boost the signal the ear receives”). Full descriptions used in each condition are displayed in Table 1 and Table 2.

### 2.3. Experimental Measures

Below the descriptions of the three fictitious hearing aid models, the website asked, “How likely would you be to purchase each model?”. Participants used a slider on a visual analog scale to rate purchase likelihood for the QX50, the QX70, and the QX90 models. The anchor points on the scale for the three models were “Very unlikely” (0) and “Very likely” (100). A number was displayed above the scale to inform participants where along the line they had set the slider arrow. Participants also used the same kind of scale to indicate their strength of agreement with the statement “I would not purchase one of the above models”, (hereafter referred to as ‘would not purchase’) with the anchors “Strongly disagree” (0) and “Strongly agree” (100). This measure was included to help emulate authentic decision-making scenarios, where consumers have the choice not to choose, and where they frequently experience indecision about whether to choose from the available options [31].

Following the decision-making scenario, the next page of the website presented participants with six statements and asked them to use visual analog scales to provide ratings that best described themselves. These statements, and their scalar anchor points, were: “I consider myself knowledgeable about hearing aids” (Strongly disagree (0)/Strongly agree (100)), “Hearing aids are important to me” (Strongly disagree (0); Strongly agree (100)), “I trust health information I read on the internet” (Strongly disagree (0); Strongly agree (100)), “I think my hearing in general is…” (Bad (0); Excellent (100)), “I think my hearing in quiet is…” (Bad (0); Excellent (100)), and “I think my hearing in background noise is…” (Bad (0); Excellent (100)). Participants were also asked to answer ‘yes’ or ‘no’ to the question “Are you currently using hearing aids to treat a hearing loss or in the process of purchasing hearing aids?”. We included these questions because previous research has shown that knowledge and personal relevance may moderate anchoring, e.g., [18,29,32] and framing effects, e.g., [33,34], trust in online information predicts purchase intention, e.g., [35,36], and the severity of hearing loss predict willingness to adopt HAs, e.g., [37].

Finally, it is common practice to include attention-checking questions in studies using MTurk workers [38]. Directly beneath the visual analog scales where participants rated the likelihood that they would purchase each hearing model, we asked participants the following attention check question: “We realize that it can sometimes be taxing to read all the details of a question and to provide a meaningful response. The purpose of this item is to assess whether you are paying attention to this question. If you are paying attention, please respond with the last response option in the list below”. The options presented below were “Male”, “Female”, and “I select this option”. Participants who failed to select “I select this option” were deemed to have failed the study’s attention checking requirement and were excluded from the dataset prior to analysis.

## 3. Results

Three hundred forty-one participant records were collected in total. Seventy (20.5%) were removed from the dataset for failing the attention checking question, leaving 271 records that met inclusion requirements for analysis. We inspected the remaining data for outliers using a *z*-score transformation. Outlying data points, with standardized values equal to or greater than +/− 2.0 [39], were found only for the QX50 and QX70 and were removed from the dataset (32 data points, or 3.05% of the total data points for dependent variables, were removed). Following the removal of outliers, skewness in excess of an absolute *z*-value of 3.29 was observed for the QX50 (−0.741, *SE* = 0.155, |*z*| = 4.78), QX70 (−0.779, *SE* = 0.155, |*z*| = 5.02), and QX90 (−0.884, *SE* = 0.148, |*z*| = 5.70), which, given the size of the samples, suggests that these distributions should be considered non-normal [40]. To meet requirements for parametric analysis, we performed a square root transformation on these variables [41], which reduced skewness (QX50 = 0.005, *SE* = 0.155, |*z*| = 0.03; QX70 = −0.066, *SE* = 0.155, |*z*| = 0.43; QX90 = 0.300, *SE* = 0.155, |*z*| = 1.94) and led to distributions that, in each case, better approximated normality.

### 3.1. Demographic Information

Of the 271 total participants, 264 (97.4%) were residents of the United States and 7 (2.6%) were residents of Canada. Among them, 138 identified as female (50.9%) and 133 identified as male (49.1%). English was reported as the primary language spoken at home by 267 (98.5%), while 1 reported Cantonese, 1 reported French, 1 reported Tamil, and 1 reported English and Spanish. More than half the sample (54.2%) reported a Bachelor’s degree as their highest level of education completed, followed by a Master’s degree or higher (24.4%). The vast majority reported their employment status as employed full time (80.1%) and their marital status as married/common law (76.7%). The most common household income range was USD 50,000–74,999 (31.0%), followed by USD 75,000–99,999 (22.5%). In total, 184 (67.9%) of participants stated that they were not currently using or in the process of purchasing HAs to treat a hearing loss; the other 87 participants (32.1%) indicated that they were. The mean age of the sample was 51.9 (*SD* = 4.88) years. A full breakdown of demographic categories is displayed in Table 3.

### 3.2. Main and Interaction Effects of Framing and Anchoring

**Figure 2:**
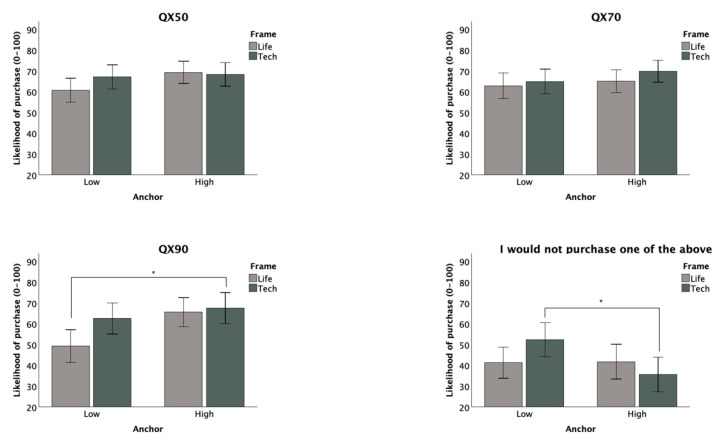
ANCOVA comparisons by group (error bars: 95% confidence intervals). * indicates a significant difference at *p* = 0.05.

For our primary analysis, we conducted four 2 (attribute framing: positive vs. negative) × 2 (anchor points: high vs. low) analyses of covariance (ANCOVA) to test the effects of the attribute framing messages and anchors on likelihood of purchasing the QX50, QX70, QX90, or none of the above. We used the square root transformations for the QX50, QX70, and QX90 in these calculations. Our six covariates were participants’ subjective ratings of knowledge about HAs, the importance HAs held for them, their trust in online health information, and their ability to hear in general, in quiet, and with background noise. After a Bonferroni adjustment to the ANCOVAs’ alpha level to control the error rate for four mean comparisons (adjusted α = 0.05/4 = 0.0125), the results did not show significant main effects for anchoring or framing for any of the four outcome measures. No significant interaction effects for anchoring and framing were shown for these measures either. Untransformed means for each condition are listed in Table 4 below. Full ANCOVA results are displayed in Table 5, and comparisons between groups for each dependent variable are displayed graphically in Figure 2 below.

There was a significant effect for the covariate ‘I consider myself knowledgeable about hearing aids’ in the model for the QX70 (*F*(3, 245) = 37.30, *p* = <0.001, *ηp^2^* = 0.132) and for the QX90 (*F*(3, 261) = 14.28, *p* = <0.001, *ηp*^2^ = 0.052). A similar effect was observed for the QX50 (*F*(3, 245) = 6.28, *p* = 0.013, *ηp*^2^ = 0.025) and for ‘would not purchase’ (*F*(3, 261) = 4.875, *p* = 0.028, *ηp*^2^ = 0.018), but these *p* values are slightly above the adjusted threshold for significance of 0.0125. An ANOVA with knowledge as the dependent variable showed no significant differences as a function of anchoring condition (*F*(3, 267) = 1.617, *p* = 0.0205, *ηp*^2^ = 0.006), framing condition (*F*(3, 267) = 2.40, *p* = 0.123, *ηp*^2^ = 0.009), or anchoring × framing (*F*(3, 267) = 3.203, *p* = 0.075, *ηp*^2^ = 0.012), indicating that the distribution of responses was not significantly different between any conditions in the study. There was also a significant effect for the covariate ‘I trust health information I read on the internet’ in the model for the QX90 (*F*(3, 267) = 7.58, *p* = 0.006, *ηp*^2^ = 0.028). As with knowledge, an ANOVA with trust as the dependent variable showed no significant differences as a function of anchoring (*F*(3, 267) = 1.233, *p* = 0.268, *ηp*^2^ = 0.005), framing (*F*(3, 267) = 2.211, *p* = 0.138, *ηp*^2^ = 0.008), or anchoring × framing (*F*(3, 267) = 0.791, *p* = 0.375, *ηp^2^* = 0.003). The means of each covariate across study conditions and Spearman’s correlations between each covariate and purchase likelihood across conditions for each untransformed dependent variable are displayed in Table 6 and Table 7 below.

### 3.3. Exploratory Analyses

In addition to our primary analysis, we conducted a series of exploratory analyses to further investigate and understand the nuances of our dataset. These analyses, while not pre-specified, offer valuable insights into potential trends and relationships that, we believe, warrant further investigation. While the findings from these explorations should be interpreted with caution, they provide intriguing directions for future research. For all the exploratory analyses detailed below, we used the same square-root-transformed versions of the QX50, QX70, and QX90 that were used in the main analyses. The means reported below use un-transformed values for ease of reader comparison. All sub-group distributions in the following analyses have absolute skewness measures that meet the specifications described by Kim [40], indicating that the shape of the distributions approximates normality.

#### 3.3.1. Exploring the Role of Self-Rated Hearing Ability in Likelihood of Purchase

**Figure 3:**
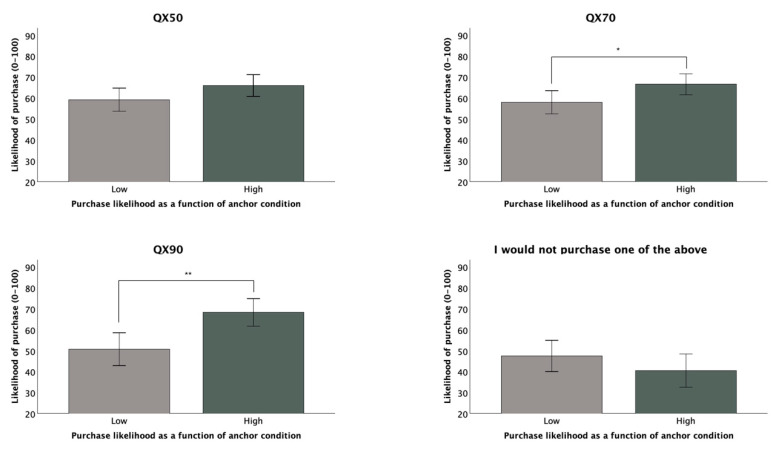
Purchase likelihood as a function of anchor condition among participants below the self-rated ‘hearing in general’ median (Error bars: 95% confidence intervals). * indicates a significant difference at *p* < 0.05, ** at <0.01.

Previous research has suggested that anchoring and framing effects may be stronger when the issue being judged is more personally relevant to participants [29,34]. Given that we recruited our sample via MTurk without specific selection for hearing capacity, we wondered whether the selective recruitment of individuals with identified or suspected hearing deficits might have yielded divergent results. Upon restricting our analyses of covariance to participants (*n* = 137) whose self-rated ‘hearing in general’ was at or below the sample’s median value (*Md* = 78), we saw a significant anchoring effect for both the QX70 (*F*(3, 117) = 4.818, *p* = 0.030, *ηp*^2^ = 0.040) and for the QX90 (*F*(3, 130) = 7.241, *p* = 0.008, *ηp*^2^ = 0.052), but no significant effect for message framing. After collapsing across messaging groups to compare by anchoring condition alone, we saw that purchase likelihood was higher in the high anchor condition for both the QX70 (*M_high_* = 66.61, *SD* = 19.94; *M_low_* = 58.03, *SD* = 21.78), *t*(117) = 2.413, *p* = 0.017, *d* = 0.433, and the QX90 (*M_high_* = 68.36, *SD* = 26.09; *M_low_* = 50.79, *SD* = 33.87), *t*(135) = 3.20, *p* = 0.002, *d* = 0.549. Figure 3 displays group differences for all outcome measures. The covariates included in the above models were participants’ self-rated knowledge of HAs, personal importance of HAs, and trust in online health information. Group means for our other two outcome measures, the QX50 and ‘would not purchase’ were not significantly different, but the nature of the differences between likelihood scores in the low and high anchoring conditions groups nevertheless appeared to dovetail with the trend described above. The high anchor group expressed greater purchase likelihood for the QX50 (*M* = 66.02, *SD* = 20.08) than the low anchor group (*M* = 59.27, *SD* = 22.67), while the high anchor group expressed weaker agreement with the statement ‘I would not purchase one of the above models’ (*M* = 40.57, *SD* = 31.76) than the low anchor group (*M* = 47.59, *SD* = 32.29), suggesting reduced resistance to the HA options we presented. Notably, we found no significant differences between conditions when repeating these analyses with the subset whose self-rated hearing in general was above the median.

#### 3.3.2. Exploring the Role of Knowledge in the Likelihood of Purchase

**Figure 4:**
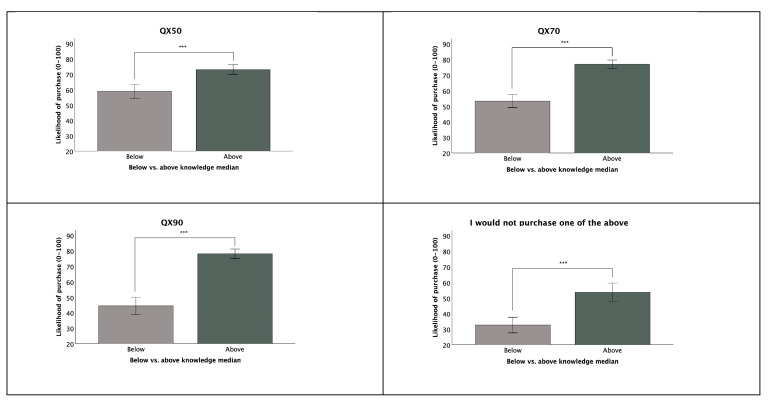
Purchase likelihood comparing participants above vs. below the self-rated HA knowledge median (error bars: 95% confidence intervals). *** indicates a significant difference at *p* < 0.001.

As HA knowledge was a significant covariate in our primary analyses, we further explored its role in purchase likelihood for each of the three devices by dividing the sample into two groups, high and low HA knowledge, based on whether participants’ self-rating was above or below the median (*Md* = 65). We conducted independent t-tests comparing high and low HA knowledge groups in terms of their purchase likelihood of each device. We found statistically significant differences between the two groups for each device, where the high HA knowledge group expressed higher purchase likelihood than the low HA knowledge group: QX50 (*t*(253) = 4.561, *p* = <0.001, *d* = 0.572); QX70 (*t*(253) = 9.428, *p* = <0.001, *d* = 1.182); QX90 (*t*(269) = 9.962, *p* = <0.001, *d* = 1.211). A statistically significant difference was also observed between the two groups for the outcome ‘I would not purchase one of the above,’ with those in the high HA knowledge group agreeing more strongly with this statement than those in the low HA knowledge group (*t*(269) = 5.378, *p* = <0.001, *d* = 0.653). This result appears to diverge from the trend among the three devices, where higher knowledge was predictive of purchase likelihood. The distribution of responses to this statement was skewed toward ‘disagree’ in the low HA knowledge group, while it was U-shaped in the high HA knowledge group. The mean comparisons for each outcome are depicted in Figure 4. Means and standard deviations for each outcome for the two groups are displayed below in Table 8.

As the sample is a heterogenous group of HA users (32.1%) and non-users (67.9%), we ran the same analysis after splitting the data by HA use or non-use and then splitting HA users and non-users into high and low HA knowledge groups using their respective median HA knowledge ratings, 83.0 and 30.0. As in the above exploratory analyses, we used the same square-root-transformed variables that were used in the main analysis. For HA users, we found statistically significant differences, in the same direction, between high and low HA knowledge groups (above or below the self-rated knowledge median of 83.0, respectively) for all three devices: QX50 (*t*(84) = 3.156, *p* = 0.002, *d* = 0.681; QX70 (*t*(83) = 2.820, *p* = 0.006, *d* = 0.612; QX90 (*t*(85) = 3.156, *p* = 0.015, *d* = 0.533. No statistically significant difference was found, however, between the two groups’ level of agreement with the statement “I would not purchase one of the above models” (*t*(85) = 0.762, *p* = 0.448, *d* = 0.163). For HA non-users, we found statistically significant differences, between high and low HA knowledge groups (above or below the self-rated knowledge median of 30.0, respectively) for two devices: the QX70 (*t*(168) = 5.457, *p* = <0.001, *d* = 0.838) and the QX90 (*t*(182) = 5.199, *p* = <0.001, *d* = 0.767). These differences were in the same direction as we found for the hearing aid users. No statistically significant difference was found, however, between the two groups’ purchase intention for the QX50 (*t*(167) = 1.246, *p* = 0.214, *d* = 0.192) or level of agreement with the statement “I would not purchase one of the above models” (*t*(182) = 1.358, *p* = 0.176, *d* = 0.200). Means and standard deviations for each outcome by knowledge group for HA users and non-users are displayed below in Table 9 and Table 10.

Additionally, as previous research has suggested that greater knowledge of the item being rated may mitigate anchoring effects, e.g., [18,32], we ran exploratory analyses of covariance for each of our four outcomes with both the above and below knowledge median subsets. Neither anchoring nor framing manipulations were significant in the models with our higher HA knowledge subset. However, for the low HA knowledge subset, anchoring was significant for the QX90 (*F*(3, 129) = 6.75, *p* = 0.01, *ηp*^2^ = 0.050) and for ‘would not purchase’ (*F*(3, 129) = 6.68, *p* = 0.002, *ηp*^2^ = 0.070). After collapsing across HA knowledge groups to compare according to the anchoring condition alone, we saw that purchase likelihood was higher in the high anchor condition for the QX90 (*M_high_* = 53.15, *SD* = 32.75; *M_low_* = 37.55, *SD* = 31.88), *t*(136) = 2.748, *p* = 0.007, *d* = 0.471, and lower in the high anchor condition for ‘would not purchase’ (*M_high_* = 23.92, *SD* = 25.09; *M_low_* = 39.82, *SD* = 31.73), *t*(136) = 3.20, *p* = 0.002, *d* = 0.549). Importantly, though, while no anchoring effect was present among the higher HA knowledge group, their mean purchase likelihood ratings for the QX90 were considerably higher than the low knowledge group in both anchoring conditions (*M_high_* = 78.88, *SD* = 17.13; *M_low_* = 77.76, *SD* = 18.14) and considerably higher for ‘would not purchase’ in both conditions (*M_high_* = 52.19, *SD* = 34.67; *M_low_* = 55.41, *SD* = 34.02).

## 4. Discussion

In this study, we explored whether individuals’ self-reported likelihood of purchasing HA options with varying levels of technology would vary based on two factors: (1) anchoring: whether participants were exposed to either a high or low price for HAs in an online banner advertisement, and (2) messaging: whether the descriptions for the range of HA options highlighted lifestyle benefits or technological capabilities. We also included several measures as covariates in our analysis—knowledge about HAs, personal importance of HAs, trust in online health information, and perceived ease of hearing in general, in quiet, and in noisy environments—which we considered to be relevant to people’s decision making when considering purchasing HAs. Following our correction for multiple comparisons, our main analyses revealed neither a significant interaction effect between anchoring and messaging nor main effects for either factor on any of our four outcomes of interest. However, our exploratory analyses revealed significantly greater purchase likelihood for two HAs in the high anchor condition among participants whose self-rating of their hearing in general was at or below the sample’s median, whereas no such differences were present among the better-hearing subset. Furthermore, we found that participants whose self-rated HA knowledge was above the sample’s median expressed significantly greater HA purchase likelihood than those whose ratings were below it. Here, we consider why our data looked the way they did and what this means for practice and future research.

### 4.1. The Role of Anchoring in Purchase Intention

In our primary analyses, we observed *p* values of 0.171, (QX50), 0.356 (QX70), 0.016 (QX90), and 0.014 (‘would not purchase’), with the latter two just missing the threshold for statistical significance when applying our adjusted alpha value of 0.0125. In our exploration of the poorer-hearing subset, we observed *p* values of 0.235 (QX50), 0.30 (QX70), 0.008 (QX90), and 0.086 (‘would not purchase’). Had our primary analyses been limited to the poorer hearing subset to begin with, only the difference observed for the QX90 would have been deemed statistically significant. It could be argued that these exploratory results are simply a similar set of response patterns with an unmanaged Type I error rate. However, we consider it important to note that these patterns disappear entirely when analyzing only the subset whose self-rated hearing in general was above the median. In other words, the poorer-hearing subset may have meaningfully contributed to the trend toward purchase likelihood differing as a function of anchoring condition in the full sample, particularly for the QX90. Additionally, the significant outcomes in the poorer-hearing subset had larger effect sizes (QX70 *ηp*^2^ = 0.040; QX90 *ηp*^2^ = 0.052) than those in our primary analysis (QX90 *ηp*^2^ = 0.022; ‘would not purchase’ *ηp*^2^ = 0.023). While the effect sizes for the exploratory outcomes may still be deemed small-to-moderate [42], they nevertheless underscore the larger impact that our anchoring had among the poorer-hearing subset.

Why was an anchoring effect present for those with poorer self-rated hearing, but not for the better group? It may be suggested that our price anchors constituted information that participants who rated their hearing lower perceived as being more important or relevant and therefore paid closer attention and were accordingly more swayed by in their likelihood of purchase ratings. However, the poorer-hearing group’s mean rating for “hearing aids are important to me” was 56.0 (*SD* = 26.24), slightly below that of the better-hearing group’s rating of 60.77 (*SD* = 30.83). Moreover, across the sample, ratings for hearing in general showed almost no correlation with HA importance, *r*(269) = 0.089, *p* = 0.143.

A further explanation for the nature of the role that anchoring played within the data may lie with the relationship between self-rated knowledge and self-rated hearing. Previous research has suggested that anchoring effects are stronger when issue knowledge is lower [18,32]. Our exploratory analyses using only the below-median knowledge subset showed significant anchoring effects for the QX90 and ‘would not purchase,’ which were the same outcomes with *p* values just below our adjusted alpha level in our primary analysis. Once again, the effect sizes for these particular comparisons (QX90 *ηp*^2^ = 0.050; ‘would not purchase’ *ηp*^2^ = 0.070) were moderate but outstripped those observed in the primary analysis, highlighting the greater impact that anchoring had on the below-median knowledge group and suggesting that the below-median knowledge subset may too have meaningfully contributed to the trend toward purchase likelihood differing as a function of the anchoring condition in the full sample.

The significant influence of anchoring among the poorer-hearing and less knowledgeable subsets raises questions about the sub-subset (*n* = 61) of participants who share both characteristics. Interestingly, when repeating our analyses only with this group, we found no significant results for anchoring in the model for any of our four outcomes. At present, then, the role that anchoring plays in influencing purchase likelihood among those with worse perceptions of their own hearing is not easily accounted for. With respect to this sample, the influence of anchoring appears independent of the role of self-perceived knowledge. The nature of the relationship between these two factors, as they relate to potential anchoring effects, warrants further investigation.

### 4.2. The Role of Knowledge in Purchase Intention

While we did not find any significant effects for anchoring/framing in the current study, a noteworthy finding was the important role that self-rated knowledge of has played in both our main and exploratory analyses. As highlighted in Table 3, knowledge was a statistically significant covariate in the ANCOVA model for each of the 4 outcome measures. Our exploratory analyses showed that higher levels of knowledge predicted purchase intention for all three devices, both in the overall sample (see Table 8 and Figure 4) and for HA users as individual groups (see Table 9). The trend was similar for HA non-users, where higher HA knowledge predicted purchase intention for the QX70 and QX90. As mentioned in Section 3, we used knowledge medians unique to the user and non-user group to run these comparisons, given that the mean level of HA knowledge among users (*M* = 81.18, *SD* = 12.15) was more than twice that of non-users (*M* = 38.47, *SD* = 32.20). It is striking, then, that higher levels of subjective knowledge among non-users were still predictive of purchase intention for two devices, even though their overall level of knowledge was low relative to users. Strong positive Spearman’s correlations, particularly between knowledge and the QX70 and QX90, further elucidate this relationship (see Table 7). They show a linear relationship between knowledge and purchase likelihood, with the highest levels of purchase likelihood expressed by those with the most knowledge (see means in Table 9 for those HA users above their knowledge median, for instance).

It is possible that participants who expressed greater HA purchase likelihood in response to the decision-making scenario may have tended to be people who already had experience with HAs or were considering purchasing them, and who felt more knowledgeable about the topic for those reasons. However, even among HA non-users, the significantly higher purchase likelihood for the QX70 and QX90 observed for those above their subset’s knowledge median still held when controlling for self-rated hearing ability and personal importance of HAs. This underscores that greater knowledge of HAs was associated with increased likelihood of purchase in our scenario, independent of personal relevance. This finding highlights the need for further research exploring the influence of HA knowledge on treatment decision-making. Researchers should consider what types of information or communication strategies most effectively contribute to decision makers’ subjective sense of HA knowledge.

Another notable point regarding knowledge is that the most knowledgeable subgroup in the sample (HA users above their group’s knowledge median) expressed higher mean purchase intention for the premium device (*M* = 82.48, *SD* = 15.43), the QX90 priced at USD 3300, than for QX70 priced at USD 2600 (*M* = 79.17, *SD* = 17.22) or the QX50 priced at USD 1900 (*M* = 77.57, *SD* = 19.27). While the present data are insufficient to demonstrate that higher levels of subjective knowledge predict preference for premium levels of technology, future research may wish to further explore this relationship.

Finally, our separate exploratory analyses of covariance with the above- and below-median knowledge subsets revealed significant anchoring effects for the QX90 and ‘would not purchase’—the same significant effects noted in our primary analyses. As these effects were not present in the subset above the knowledge median, it appears as though the below-median group’s response may have been driving the effects that were observed in the main analyses. As noted earlier, finding anchoring effects among those with lower knowledge is in line with earlier findings in the literature, which suggest an inverse relationship between the strength of anchoring effects and knowledge of the issue, e.g., [18,32].

### 4.3. Why Was the Effect of Framing Not Stronger?

Our results clearly support the notion that our messaging manipulation had no influence on participants’ purchase intention. Below, we consider the merits of three potential explanations: whether our HA importance covariate accounted for variance that might otherwise have been attributed to framing, whether our attribute frames were different enough from one another, and whether participants may not have deeply engaged with the product descriptions.

As for controlling for HA importance, participants’ responses to “Hearing Aids are important to me” served as a measure of ‘issue relevance’, which has been shown to moderate the influence of framing effects, e.g., [43,44]. However, while HA importance showed significant moderately strong positive correlations with QX70 and QX90 purchase intention (see Table 7), it was not a significant covariate in our ANCOVA models (see Table 3), nor did exploratory analyses show any noteworthy difference in purchase intention for the three devices when comparing participants above and below the median rating for HA importance. Further exploratory analysis showed that framing was neither predictive of purchase intention for the three devices among HA-users, whose mean HA importance rating was 79.90 (*SD* = 10.67), nor among non-users, whose mean HA importance rating was 48.17 (*SD* = 28.85). This suggests that controlling for HA importance did not account for variance in our models that otherwise may have been attributed to framing effects.

Regarding differences between attribute frames, a second potential reason for the lack of observed effects for framing could be that the lifestyle-emphasizing and technology-emphasizing product descriptions were not sufficiently different from one another to make differential impressions upon participants. The key rhetorical differences between the two conditions were that the lifestyle-emphasizing description used more personal language (including frequent use of ‘you’ and ‘your’) and highlighted how the devices would improve daily living experiences, while the tech-emphasizing description used impersonal language and focused on how the features worked. The following passages illustrate these differences: “The Mask Mode and StereoZoom features are designed with noisy and physically distanced environments in mind, allowing you to continue hearing as clearly as possible in challenging situations” (lifestyle) versus “Mask Mode uses artificial intelligence to boost speech clarity in difficult listening situations. StereoZoom makes it easier for the ear to differentiate between speech coming from multiple directions, by focusing on target sounds” (technology) (see Table 1 and Table 2 for full product descriptions). However, both conditions’ descriptions highlighted features of all three devices, meaning that the lifestyle condition still contained references to the technology despite its more personalized emphasis on benefits to daily activities. Furthermore, both conditions provided the same checklist of available features for each device. Future research may wish to gather qualitative perceptions of divergent HA device descriptions or empirically explore attitudes and intentions in response to HA descriptions that bear more overt differences to one another. It may be the case, though, that artificially divergent descriptions would sacrifice ecological validity: we have anecdotally observed that descriptions of hearing aids online tend to simultaneously incorporate descriptions of technological features and lifestyle benefits, and we attempted to recreate the style of these descriptions in our study materials.

In terms of engagement with product descriptions, a third potential reason may be related to the extent to which participants engaged with the product descriptions. If we assume that there were indeed potentially meaningful differences between the lifestyle- and technology-emphasizing descriptions, then participants may have needed to engage with the descriptions to a certain degree, or in a specific manner, for these differences to have had a chance at being influential, as the differences may not have been readily apparent with only a cursory glance. The ‘degree’ and ‘specifics’ that would be necessary to initiate the hypothesized effect warrant future consideration. It also may have been the case that participants, on average, paid more attention to the list of features for each device (which were identical across conditions) or the retail prices. The use of eye-tracking measures to gain insight into how consumers process product information online has been growing, e.g., [45,46]. Incorporating eye-tracking into a study such as this one would contribute to a fuller understanding of which aspects of the product descriptions we provided captured readers’ attention; it may permit further insight into whether rhetorical differences like the ones we employed are meaningful in the online contexts where prospective HA buyers access product information.

### 4.4. Implications

The impetus for this study was to gain insight into whether encountering low OTC HA prices online may influence purchase intention for HAs at a range of technological levels, with prices and features that are representative of those that would be prescribed in a brick-and-mortar hearing clinic. In addition to the potential impact of exposure to low prices, we also wanted to know what impact exposure to high prices might have, as well as whether differences in device descriptions may play a role. While we did not observe significant effects for message framing, we did find that exposure to high or low prices prior to a decision-making scenario about purchasing HAs had a significant influence on HA purchase likelihood among the portion of our sample whose self-rated hearing fell at or below the median for the group. Namely, we observed that, among those with poorer self-rated hearing, those who saw an advertisement featuring high-priced devices expressed significantly greater purchase likelihood for mid-range and premium HAs than did those who saw an ad with low-priced devices. This suggests that potential buyers who see sponsored advertisements quoting low prices may be less willing to purchase hearing aids with higher levels of technology than potential buyers who see similar advertisements quoting higher prices.

As our study did not include a control condition, we cannot determine the extent to which these response patterns differ from what we would have observed in the absence of an anchoring manipulation. While we predict that a control group’s mean ratings would have been in between those observed in our high and low anchor conditions, we do not know which anchoring condition exerted the stronger sway on participants’ responses within our present sample. Nevertheless, our results provide grounds to suspect that the prices that HA candidates encounter in one context may have the potential to influence their perceptions of the prices they encounter in another. We believe this area of research warrants continued attention: while prescription HAs are not currently sold directly to consumers online, growing trends toward telehealth (including teleaudiology) provision suggest that a fuller understanding of the factors that affect potential HA buyers’ purchase intentions may be fruitful.

Beyond anchoring, we wish to emphasize the importance of the association we observed between self-rated knowledge and purchase likelihood, particularly for devices with higher levels of technology. Outcomes for the QX90, our premium device priced at USD 3300, make for a useful demonstration of this point: among non-HA users, median self-rated knowledge of HAs was just 30 on a scale of 0 to 100. Those below this mark rated their purchase likelihood of the QX90 at an average of 39.97 out of 100, while those above the mark rated their purchase likelihood 63.6% higher, at an average of 65.37 out of 100. In other words, even the perception that one’s HA knowledge is moderate is associated with an appreciable increase in purchase intention in comparison with those who believe their HA knowledge to be low. The contrast is even starker when examining the whole dataset and comparing those below with those above the grand median of 65: QX90 purchase likelihood for those below averaged 44.44; for those above, it averaged 78.32, or 76.2% higher.

While we have described how exposure to a high anchor increased purchase intention when examining both those with self-rated hearing and self-rated knowledge below their respective medians, the magnitude of the increases associated with high anchors (from 50.79 to 68.37, or +34.7%, and from 37.55 to 53.15, or +41.5%, for the QX90 for the poorer hearing and less knowledgeable subsets, respectively) is less than the magnitude of the increase associated simply with higher self-perceived knowledge, as described above. So, while the potential deployment of heuristic strategies including anchoring may be of interest to HA manufacturers or vendors, these findings suggest that investing in strategies for effective HA education may be the most worthwhile pursuit in the effort to address ongoing low rates of HA uptake [47]. The significant influence of anchoring we observed in the less knowledgeable subset calls attention to the value of investing in HA education as a strategy to mitigate the potential influence of the types of low prices commonly featured in the advertisements we mentioned at the outset.

Finally, it is worth noting that the above implications may apply more directly to the United States than to other markets. Some countries prohibit advertising medical products or offer state-sponsored reimbursement plans for hearing aids, making cost a less significant consideration for potential users.

### 4.5. Limitations

There are some aspects of our sample and design that may limit the generalizability of our findings. First, our sample predominantly comprised people who were married, full-time employed, college educated, and younger than the typical age at which Americans adopt HAs, though not younger than the age at which many individuals with hearing loss begin considering using HAs [48]. Apart from the relatively young mean age, these characteristics have each been shown to be associated with an increased likelihood of adopting HAs [49]. These demographic factors suggest that the levels of purchase intention we report may not be representative of those that would have been observed among a sample with different characteristics. Additionally, our sample was not limited to individuals who are already aware of a hearing loss. Future research may wish to explore similar questions with a more targeted sample, such as individuals with a specific hearing loss profile.

Second, while we asked participants to rate their perceptions of their own hearing, we did not collect clinical audiological data. Severity of hearing loss, as measured in clinics, is the most frequently documented predictor of hearing aid uptake in the literature [49]. With the current sample, we are unable to comment on what role degree of participant hearing loss may have played in our analyses. Third, our study design did not include a control group, which limits our ability to interpret the extent to which our observed exploratory anchoring effects diverge from the purchase intention participants would have expressed in the absence of price exposure prior to our rating scenario.

Third, our data were collected on Amazon’s Mechanical Turk platform, whose users may have a higher level of familiarity with common experimental manipulations. While MTurk users have been shown to produce reliable data that are consistent with more traditional sample sources [50], they have also been shown to be especially attentive to attention checking and manipulation checking questions [51]. As described in Section 3, we discarded participants who failed our attention checking question. Of the 271 participants who remained in our sample, 231 responded “yes” to a manipulation-checking question at the end of the study that asked “did you notice the advertisements for hearing aids during the first section of the study?”. Without evidence about the level of attention paid (e.g., eye tracking measures), it is difficult to discern whether noticing the ads at such high rates may have contributed to or diminished the effect of anchoring manipulations. However, the high rate of affirmative responses to this question indicates that the vast majority of our participants were likely paying close attention. As such, this sample’s responses may not be representative of the amount and intensity of attention paid by the public when looking for hearing aid information online, whose attention may range from being divided due to online multi-tasking to being even more highly focused if their search holds high personal relevance.

Fourth, while our data suggest that high anchors tend to increase purchase intention of HAs in comparison with low anchors, this study presented its anchors as though they were prices for one fictitious HA company, and then asked participants to rate their purchase intention for devices from a different fictitious company. This arrangement may mean that our observed outcomes were the product of participants engaging in ‘comparison shopping’ between brands, and thus they may not inform advertising strategies for manufacturers aiming to persuade potential buyers to purchase their own product.

Finally, we acknowledge that our use of exploratory analyses may raise concerns about Type I error rate. We recommend interpreting these results with caution, and we view the findings we present here as a first step toward further research in this area. Subsequent studies asking similar questions may be strengthened by pre-registering plans for data analysis.

## 5. Conclusions

This study aimed to understand whether online exposure to different HA prices and differently framed device descriptions would influence purchase intentions. The current findings suggest that, among individuals with poorer perceptions of their own hearing, exposure to high or low HA prices may influence their likelihood of purchasing devices within typical prescription price ranges, supporting the theory that anchoring effects are stronger among those with less issue knowledge. Furthermore, our data shed light on the power of perceived knowledge about HAs: people who feel they know more about hearing aids indicate that they have greater intention to purchase hearing aids. Thus, efforts to educate potential buyers could be instrumental in driving sales, addressing the larger issue of low HA uptake. These findings should not be seen as an end, but rather a starting point for future research. They underscore the need for continued study, especially as teleaudiological options continue to expand.

## Figures and Tables

**Table 1:** Hearing aid descriptions used in the lifestyle-focused condition.

Model	Description	Features	Price
QX50	The QX50 will help you hear better and will allow you to more easily enjoy sound in your daily life. For instance, the radio in your car and conversations with friends will sound louder and crisper. Feedback cancellation minimizes distracting noises, allowing you to focus on the sounds you want to hear.	✓ Directional microphone ✓ Feedback cancellation	USD 1900
QX70	The QX70 is designed with your connected life in mind. Smart device streaming lets you answer phone calls and enjoy the sound from your favorite TV shows directly through your hearing aid. The QX70 is also adjustable via an app, allowing you to customize your listening settings to suit your preferences.	✓ Directional microphone ✓ Feedback cancellation ✓ Smart device streaming ✓ Adjustable via app	USD 2600
QX90	The QX90 is the most advanced device and will help you hear your best in the widest range of today’s situations. The Mask Mode and StereoZoom features are designed with noisy and physically distanced environments in mind, allowing you to continue hearing as clearly as possible in challenging situations. These features also help reduce the effort it takes when you’re trying to listen in these environments.	✓ Directional microphone ✓ Feedback cancellation ✓ Smart device streaming ✓ Adjustable via app ✓ Mask Mode ✓ StereoZoom	USD 3300

**Table 2:** Hearing aid descriptions used in technology-focused condition.

Model	Description	Features	Price
QX50	The QX50 will help boost the signal the ear receives. The key feature is a directional microphone, which makes the target signal louder than the background noise. This device also comes with feedback cancelation to minimize squeaking sounds from the device that sometimes happen with hearing aids.	✓ Directional microphone ✓ Feedback cancellation	USD 1900
QX70	The QX70 includes all the features of the QX50 and offers the extra benefit of audio streaming with smart devices, like phones or TVs. Additionally, the QX70 is compatible with an iPhone/Android app that allows the device to be adjusted comfortably in various listening settings.	✓ Directional microphone ✓ Feedback cancellation ✓ Smart device streaming ✓ Adjustable via app	USD 2600
QX90	The QX90 offers the most advanced functionality. Including all the features of the QX70, this model also introduces Mask Mode and StereoZoom. Mask Mode uses artificial intelligence to boost speech clarity in difficult listening situations. StereoZoom makes it easier for the ear to differentiate between speech coming from multiple directions, by focusing on target sounds.	✓ Directional microphone ✓ Feedback cancellation ✓ Smart device streaming ✓ Adjustable via app ✓ Mask Mode ✓ StereoZoom	USD 3300

**Table 3:** Demographic category frequency counts.

Characteristic	Category	*N*	Percentage (%)
Highest level of education completed	Less than high school	1	0.4
	High school	9	3.3
	Trade/technical/vocational	12	4.4
	Some university/college	36	13.3
	Bachelor’s degree	147	54.2
	Master’s degree or higher	66	24.4
Employment status	Employed part time	21	7.7
	Employed full time	217	80.1
	Retired	4	1.5
	Unable to work	8	3.0
	Unemployed	18	6.6
	Other	3	1.1
Marital status	Single	30	11.1
	Married (or common law)	208	76.7
	Separated	4	1.5
	Divorced	26	9.6
	Widowed	3	1.1
Household income (in US dollars)	Under USD 25,000	26	9.6
	USD 25,000–39,999	26	9.6
	USD 40,000–49,999	43	15.9
	USD 50,000–74,999	84	31.0
	USD 75,000–99,999	61	22.5
	Over USD 100,000	31	11.4
Currently using HAs (or in the process of purchasing them)	No	184	67.9
	Yes	87	32.1

**Table 4:** Mean purchase likelihood for each device by experimental condition.

	Condition	*N*	Mean (Out of 100)	*SD*
QX50	Low/Lifestyle	65	60.77	23.15
	Low/Technology	67	67.13	23.94
	High/Lifestyle	61	69.34	20.73
	High/Technology	62	68.37	22.25
QX70	Low/Lifestyle	62	62.86	24.25
	Low/Technology	67	64.97	24.27
	High/Lifestyle	64	65.06	22.27
	High/Technology	62	69.89	20.71
QX90	Low/Lifestyle	69	49.22	33.12
	Low/Technology	74	62.53	32.09
	High/Lifestyle	64	65.61	27.82
	High/Technology	64	67.63	29.80
Would not purchase	Low/Lifestyle	69	41.28	31.00
	Low/Technology	74	52.37	35.24
	High/Lifestyle	64	41.78	33.49
	High/Technology	64	35.66	33.52

**Table 5:** Results of ANCOVA tests.

Measures	QX50		QX70		QX90		Would Not Purchase	
	*F*	*η_p_* ^2^	*F*	*η_p_* ^2^	*F*	*η_p_* ^2^	*F*	*η_p_* ^2^
Anchor	1.89	0.008	0.85	0.003	5.87 *	0.022	6.09 *	0.023
Frame	0.18	0.001	0.18	0.001	2.82	0.011	0.001	0.000
Anchor × Frame	1.10	0.004	1.77	0.007	0.26	0.001	2.54	0.010
Knowledge	6.28 *	0.025	37.30 ^‡^	0.132	14.28 ^‡^	0.052	4.88 *	0.018
Trust	0.04	0.000	0.18	0.001	7.58 *	0.028	0.14	0.001
Importance	1.51	0.002	0.07	0.000	2.78	0.011	2.50	0.009
Hearing in quiet	3.04	0.012	2.90	0.012	0.04	0.000	0.66	0.003
Hearing in noise	0.57	0.002	0.17	0.001	0.001	0.000	1.82	0.007
Hearing in general	0.29	0.001	3.20	0.013	0.45	0.002	1.12	0.004

Note: the above *F* values were calculated using square root transformed data for the QX50, QX70, and QX90, as described in the text. * *p* < 0.05, ^‡^ *p* < 0.001.

**Table 6:** Covariate means (out of 100) and standard deviations by study condition.

Covariate	Condition			
	Low/Lifestyle	Low/Tech	High/Lifestyle	High/Tech
Knowledge	42.8 (32.6)	56.4 (34.5)	55.3 (33.0)	54.3 (34.2)
Trust	58.9 (20.6)	65.6 (25.8)	64.5 (22.5)	66.2 (23.4)
HA importance	52.9 (25.4)	60.5 (28.6)	58.6 (30.4)	61.5 (30.1)
Hearing in general	73.4 (19.4)	75.1 (17.7)	76.3 (18.7)	77.9 (16.7)
Hearing in noise	66.9 (21.5)	72.6 (20.0)	71.3 (21.0)	74.6 (19.7)
Hearing in quiet	78.3 (17.8)	77.5 (18.0)	75.4 (18.5)	80.0 (17.8)

**Table 7:** Spearman’s correlations between significant covariates and purchase likelihood.

Covariate	Condition (Anchor/Frame)		Dependent Variables			
			QX50	QX70	QX90	Would Not Purchase
Knowledge	Low/Lifestyle		0.158	0.499 ^‡^	0.469 ^‡^	0.072
	Low/Tech		0.433 ^‡^	0.621 ^‡^	0.593 ^‡^	0.269 *
	High/Lifestyle		0.18	0.465 ^‡^	0.488 ^‡^	0.321 *
	High/Tech		0.399 ^†^	0.604 ^‡^	0.480 ^‡^	0.329 ^†^
Trust	Low/Lifestyle		0.115	0.300 *	0.435 ^‡^	0.175
	Low/Tech		0.484 ^‡^	0.540 ^‡^	0.583 ^‡^	0.112
	High/Lifestyle		0.023	0.351 ^†^	0.467 ^‡^	0.248 *
	High/Tech		0.269 *	0.342 ^†^	0.430 ^‡^	0.157
HA Importance	Low/Lifestyle		0.273 *	0.455 ^‡^	0.434 ^‡^	0.198
	Low/Tech		0.353 ^†^	0.474 ^‡^	0.501 ^‡^	0.148
	High/Lifestyle		−0.089	0.456 ^‡^	0.392 ^†^	0.411 ^‡^
	High/Tech		0.253 *	0.414 ^‡^	0.383 ^†^	0.308 *
Hearing in general	Low/Lifestyle		0.072	0.267 *	0.225	−0.006
	Low/Tech		0.435 ^‡^	0.498 ^‡^	0.242 *	0.018
	High/Lifestyle		0.206	−0.056	0.050	−0.007
	High/Tech		0.344 ^†^	0.262 *	0.060	−0.166
Hearing in noise	Low/Lifestyle		0.056	0.284 *	0.185	0.059
	Low/Tech		0.399 ^‡^	0.407 ^‡^	0.225	0.102
	High/Lifestyle		0.375 ^†^	0.110	0.142	0.166
	High/Tech		0.361 ^†^	0.241	0.173	−0.129
Hearing	Low/Lifestyle		0.081	0.287 *	0.008	−0.016
in quiet	Low/Tech		0.178	0.318 ^†^	0.028	−0.208
	High/Lifestyle		0.365 ^†^	−0.073	0.001	−0.106
	High/Tech		0.264 ^†^	0.210	0.026	−0.136

* *p* < 0.05, ^†^ *p* <0.01, ^‡^ *p* < 0.001.

**Table 8:** Mean purchase likelihood (out of 100) for each device below/above knowledge median.

Device	Knowledge (Above/Below Median)	*N*	Mean	*SD*
QX50	Below	123	59.03	24.71
	Above	132	73.15	18.28
QX70	Below	123	53.37	23.43
	Above	132	77.14	15.35
QX90	Below	138	44.44	33.08
	Above	133	78.32	17.58
Would not purchase	Below	138	32.79	29.95
	Above	133	53.79	34.26

**Table 9:** Mean purchase likelihood (out of 100) for each device below/above the knowledge median among HA users.

Device	Knowledge (Above/Below Median)	*N*	Mean	*SD*
QX50	Below	44	67.93	17.02
	Above	42	77.57	19.27
QX70	Below	43	72.37	10.32
	Above	42	79.17	17.22
QX90	Below	44	75.84	16.55
	Above	43	82.48	15.43
Would not purchase	Below	44	54.64	30.26
	Above	43	53.79	33.06

**Table 10:** Mean purchase likelihood (out of 100) for each device below/above the knowledge median among non-HA users.

Device	Knowledge (Above/Below Median)	*N*	Mean	*SD*
QX50	Below	83	59.63	26.86
	Above	86	66.52	20.28
QX70	Below	82	50.63	24.44
	Above	88	69.98	21.17
QX90	Below	93	39.97	33.50
	Above	91	65.37	28.08
Would not purchase	Below	93	33.19	31.85
	Above	91	39.74	33.50

## Data Availability

The dataset collected and analyzed in this study will be made available upon reasonable request.
